# Down-Regulation of Shadoo in Prion Infections Traces a Pre-Clinical Event Inversely Related to PrP^Sc^ Accumulation

**DOI:** 10.1371/journal.ppat.1002391

**Published:** 2011-11-17

**Authors:** David Westaway, Sacha Genovesi, Nathalie Daude, Rebecca Brown, Agnes Lau, Inyoul Lee, Charles E. Mays, Janaky Coomaraswamy, Brenda Canine, Rose Pitstick, Allen Herbst, Jing Yang, Kerry W. S. Ko, Gerold Schmitt-Ulms, Stephen J. DeArmond, Debbie McKenzie, Leroy Hood, George A. Carlson

**Affiliations:** 1 Centre for Prions and Protein Folding Diseases, University of Alberta, Edmonton, Alberta, Canada; 2 Department of Biochemistry, University of Alberta, Edmonton, Alberta, Canada; 3 McLaughlin Research Institute, Great Falls, Montana, United States of America; 4 Institute for Systems Biology, University of Washington, Seattle, Washington, United States of America; 5 Department of Cellular Neurology, Hertie Institute for Clinical Brain Research, University of Tuebingen, Tuebingen, Germany; 6 Centre for Research in Neurodegenerative Diseases, University of Toronto, Ontario, Canada; 7 Department of Laboratory Medicine and Pathobiology, University of Toronto, Ontario, Canada; 8 Department of Pathology, University of California San Francisco, San Francisco, California, United States of America; University of Edinburgh, United Kingdom

## Abstract

During prion infections of the central nervous system (CNS) the cellular prion protein, PrP^C^, is templated to a conformationally distinct form, PrP^Sc^. Recent studies have demonstrated that the *Sprn* gene encodes a GPI-linked glycoprotein Shadoo (Sho), which localizes to a similar membrane environment as PrP^C^ and is reduced in the brains of rodents with terminal prion disease. Here, analyses of prion-infected mice revealed that down-regulation of Sho protein was not related to *Sprn* mRNA abundance at any stage in prion infection. Down-regulation was robust upon propagation of a variety of prion strains in *Prnp* ^a^ and *Prnp* ^b^ mice, with the exception of the mouse-adapted BSE strain 301 V. In addition, Sho encoded by a Tg*Sprn* transgene was down-regulated to the same extent as endogenous Sho. Reduced Sho levels were not seen in a tauopathy, in chemically induced spongiform degeneration or in transgenic mice expressing the extracellular ADan amyloid peptide of familial Danish dementia. Insofar as prion-infected *Prnp* hemizygous mice exhibited accumulation of PrP^Sc^ and down-regulation of Sho hundreds of days prior to onset of neurologic symptoms, Sho depletion can be excluded as an important trigger for clinical disease or as a simple consequence of neuronal damage. These studies instead define a disease-specific effect, and we hypothesize that membrane-associated Sho comprises a bystander substrate for processes degrading PrP^Sc^. Thus, while protease-resistant PrP detected by *in vitro* digestion allows *post mortem* diagnosis, decreased levels of endogenous Sho may trace an early response to PrP^Sc^ accumulation that operates in the CNS *in vivo.* This cellular response may offer new insights into the homeostatic mechanisms involved in detection and clearance of the misfolded proteins that drive prion disease pathogenesis.

## Author Summary

In prion infections of the nervous system the cellular prion protein, PrP^C^, changes to a distinct form, PrP^Sc^. Recent studies have demonstrated that another glycoprotein Shadoo (Sho), which occupies a similar membrane environment as PrP^C^, is reduced in the brains of rodents with terminal prion disease. Our analyses of prion-infected mice revealed that reduction of Sho protein was not due to reductions in the corresponding messenger RNA. Reduction in Sho was clearly evident upon propagation of a variety of prion strains, but was not seen in mice with other types of neurodegenerative disease. Also, as prion-infected mice with only one copy of the PrP gene exhibited both accumulation of PrP^Sc^ and a reduction of Sho protein hundreds of days prior to onset of neurologic symptoms, the drop in Sho protein level can be excluded as an important trigger for clinical disease, or a non-specific consequence of brain cell damage. Instead, our studies define a effect restricted to prion disease and we hypothesize that Sho protein is a “bystander” for degradative processes aimed at destroying PrP^Sc^.

## Introduction

Prion diseases, including the prototypical scrapie of sheep and Creutzfeldt-Jakob Disease (CJD) of humans, are fatal and incurable neurodegenerative disorders. They are unusual in that they are often transmissible or infectious diseases. While they can be studied to great effect in a lab setting (experimental prion disease), they can also be initiated inadvertently with contaminated material. Thus, in the case of variant CJD (vCJD), occurrence is linked to the UK epidemic of Bovine Spongiform Encephalopathy (BSE) and is thought to involve infection by an oral route from BSE-contaminated food [1], [2]. In the disease process a benign, host-encoded α-helical glycoprotein (prion protein, PrP^C^) undergoes a conformational transition to a β-sheet enriched and infectivity-associated form commonly denoted PrP^Sc^ (sometimes denoted as PrP^d^). This transition is often marked by reduced detergent solubility and acquisition of resistance to proteinase K (PK) digestion *in vitro*. In strong and independent support of a central role for altered PrP in disease pathogenesis and replication of the transmissible agent, missense mutations in the murine prion protein structural gene, *Prnp*, that create the allelic forms *Prnp* ^a^ and *Prnp* ^b^ cause a modulation of prion disease phenotypes and differential susceptibility to prion strain isolates [3]–[5], while knockout of mouse *Prnp* renders animals completely resistant to experimental prion infections [6]. In addition, recombinant PrP ‘misfolded’ *in vitro* has been shown to generate prion infectivity [7], [8].

A battery of analytical techniques demonstrates that disease-associated forms of PrP are variegated. Thus, there is heterogeneity with respect to the concentration of PK needed for complete digestion, the positions of N-terminal PK cleavage sites, detergent insolubility, antibody accessibility and denaturation with guanidinium [9]–[14]. Given this biochemical heterogeneity and PrP's genetically-defined central role in disease, there have been attempts to align different facets of the disease process - latency, replication rate, infectious titre and clinical symptomatology - with the sub-varieties of PrP^Sc^ that are themselves distinct from PrP^C^ [15], [16]. Several of these biochemical heterogeneities map to a region N-terminal to PrP^C^'s hydrophobic domain (HD). Interestingly, the recently discovered Shadoo (Sho) protein bears similarity to this region of PrP by containing an HD and a preceding series of tandem repeats with positively charged residues. Like the equivalent region in PrP, this part of Sho is also considered to be natively unstructured [17]–[19].

As Sho is decreased in prion-infected brains [17] and has neuroprotective activity [17], [20] we hypothesized previously that the disappearance of Sho might contribute to the onset of clinical signs [17]. Experiments here repeal this hypothesis and instead show that down-regulation is present pre-clinically. Surprisingly, down-regulation parallels a particular biochemical signature of infection, namely appearance of protease-resistant PrP. Our cumulative data suggest a hypothesis wherein Sho comprises a “tracer” molecule that reveals a specific *in vivo* pathway with action directed against, or activated by, protease-resistant PrP^Sc^.

## Results

### Reduction of Sho levels during the course of prion infections

Prior studies of prion infection with the RML isolate demonstrated down-regulation of Sho in different cohorts of animals analyzed in the clinical phase of disease [17]. We drew upon three resources to assess the kinetics of down-regulation of the Sho protein in animals infected with the same type of agent.

**Figure 1:**
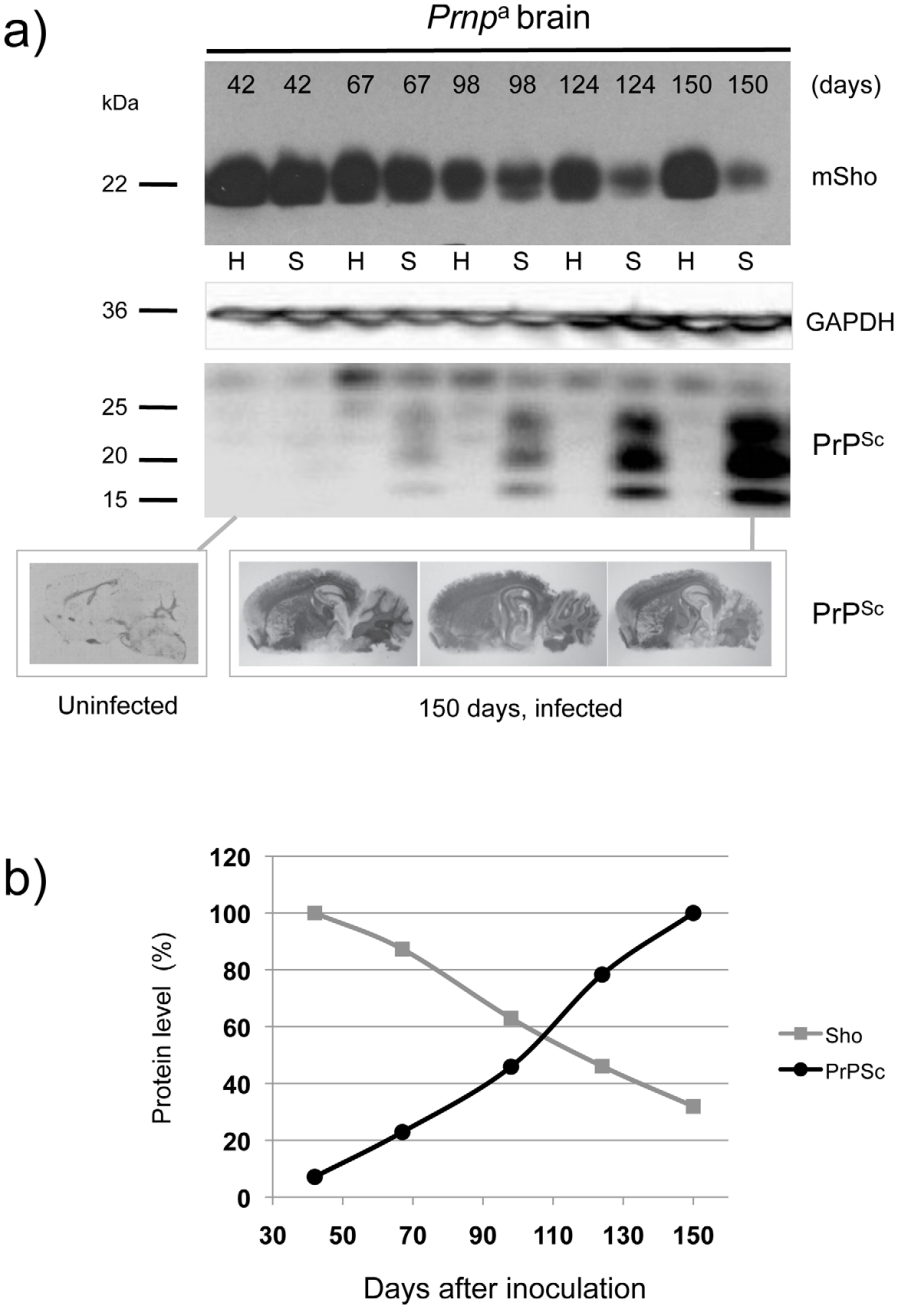
Kinetics of Sho down-regulation in scrapie-infected wt *Prnp* ^a/a^ mouse brain. ***a)*** Time-course of reduction of mature full-length 22 kDa Shadoo protein (mSho) in *Prnp* ^a/a^ mice inoculated with RML scrapie prions and analyzed at the indicated days post-inoculation (06rSH1 antibody). Pair-wise analyses are shown horizontally where “H” designates healthy negative control mice inoculated with control brain whereas “S” designates mice inoculated with the RML isolate of scrapie prions. Glyceraldehyde phosphate dehydrogenase (GAPDH) blot demonstrates similar protein loading for “H” and “S” samples. Western blot analyses show Sho levels in untreated brain samples whereas levels of PrP^Sc^ were assessed after PK digestion performed prior to sample electrophoresis and immunoblotting with D18 antibody. Brain histoblot analyses of protease-resistant PrP (D18 antibody) for three mice at the 150-day time-point are shown below the gel analysis, marked “PrP^Sc^”, to demonstrate reproducibility of the protein accumulation. ***b)*** Graphical representation of Sho and PrP^Sc^ levels in RML-inoculated animals with Sho normalized to 100% for the day 42 time-point in the preclinical phase of disease and with PrP^Sc^ normalized to 100% for the day 150 time-point in the clinical phase of disease. In both cases half-maximal change occurred approximately 50 days before the experiment endpoint and at ∼65% elapsed time in disease incubation period.

The first corresponded to wild type (wt) *Prnp* ^a/a^ mice infected intracerebrally with RML prions (scrapie, “S”) or control brains (healthy, “H”) and with brain samples isolated from early time-points onwards subsequent to inoculation (**Figure 1a**). Here, levels of full-length Sho were slightly depressed at 98 days post-inoculation and unequivocally depressed at 124 days post-inoculation, the latter time-point being one month before onset of clinical disease in these animals (150 day time-point shown here, average time to disease for this cohort is 154 days in the FVB/N background, 161 days in C57 mice [21]–[23]). As can be noted in these animals expressing wt levels of *Prnp* ^a^, the time-points at which Sho levels fall by 50% and PrP^Sc^ rise to 50% final concentration are similar (**Figure 1b**), and occur approximately 50 days in advance of clinical disease.

**Figure 2:**
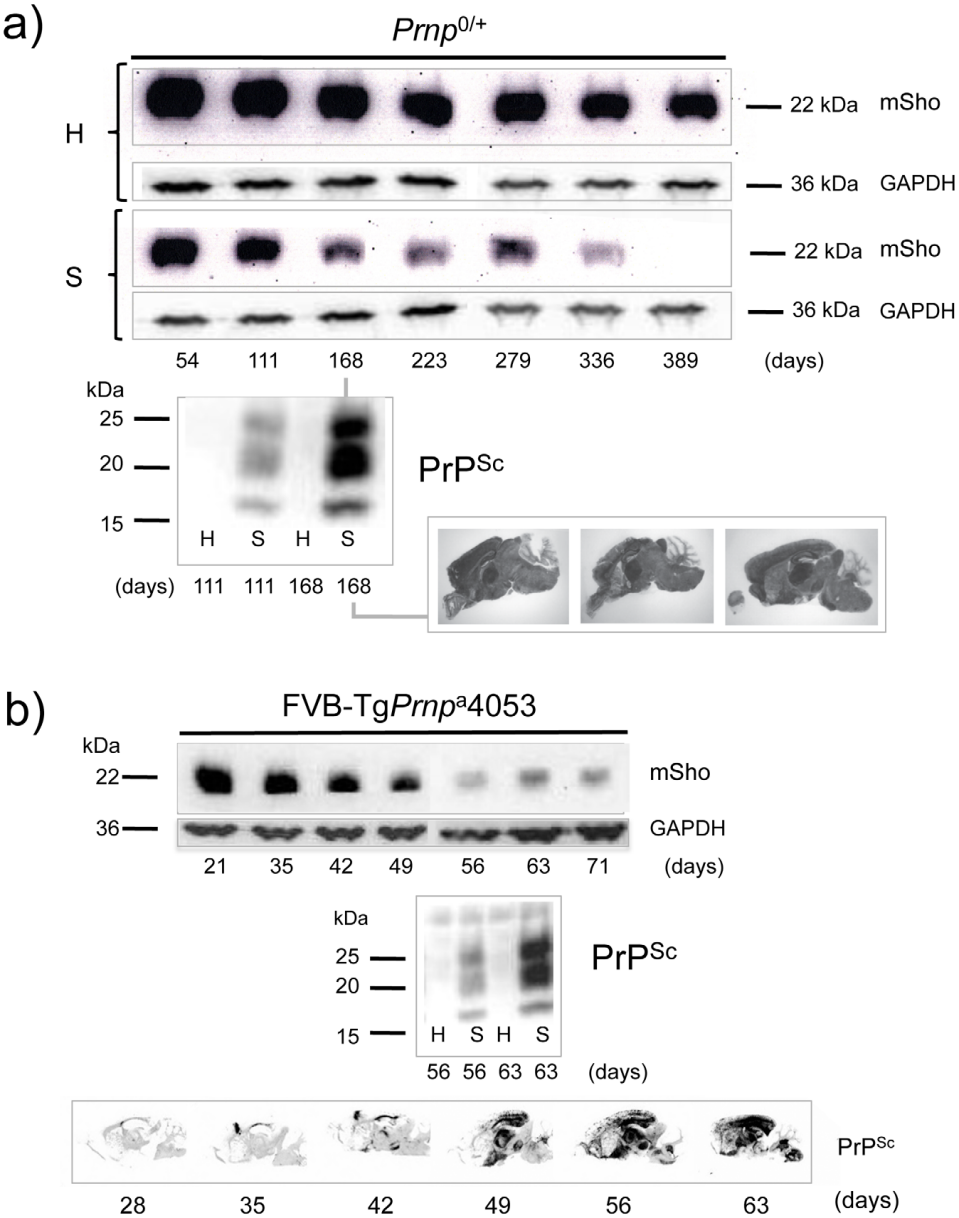
Kinetics of Sho down-regulation in mice with altered *Prnp* ^a^ gene-dosage. ***a)*** Time-course of reduction of Sho protein in mice hemizygous for *Prnp* ^a^ (*Prnp* ^0/+^). Pair-wise analyses (06rSH1 antibody) are presented vertically with the upper row representing control healthy (H) mice and the lower row representing mice inoculated with RML scrapie prions (S), and re-probing as in Figure 1. The time-course was analyzed at different time-points post-inoculation, as shown. The increment in PrP^Sc^ levels in scrapie-infected mice (S) detected by western blot analysis is shown for the 111 and 168-day timepoints, with control mice (H) analyzed alongside. Brain histoblot analyses for PrP^Sc^ in three mice at the day 168 time-point (pre-clinical phase) are shown as per Figure 1 (D18 antibody). ***b)*** Time-course of reduction of Sho protein in Tg*Prnp* ^a^4053 mice overexpressing PrP-A protein. The increment in PrP^Sc^ levels between days 56 and 63 is illustrated by western blot analysis. Histoblot time-course (lower panel) demonstrates the accelerated tempo of disease in this transgenic line. PrP analyses were with antibody D18.

A second resource corresponded to mice hemizygous for the wt *Prnp* ^a^ allele (for simplicity the *a* allele is designated by a ‘plus’ sign, “*Prnp* ^0/+^”). Prior studies have shown that the time to clinical disease is greatly extended in these animals-by hundreds of days-whereas accumulation of protease-resistant PrP^Sc^ is not linked to clinical symptoms of disease and occurs at an earlier stage [23], [24]. These earlier findings were confirmed for a cohort of hemizygous mice infected with the RML isolate of mouse-adapted scrapie, as protease-resistant PrP^Sc^ was evident in the brain of infected animals over 220 days before the endpoint when clinical signs were apparent [23], [24] (168-day time-point shown, **Figure 2a**). Notably, by examination of pair-wise permutations of control animals inoculated with non-infectious material and prion-inoculated animals, down-regulation of Sho protein was clearly apparent at the same 168-day time-point.

A third resource corresponded to transgenic (Tg) mice overexpressing the PrP-A allelic form of the PrP^C^ protein encoded by a *Prnp* ^a^ cDNA [22], with these Tg4053 mice having a greatly accelerated tempo of prion disease. Here again, Sho levels fell in advance of the mice attaining the clinical phase of disease, but with down-regulation apparent at time-points where PrP^Sc^ was readily detectable by western blot and histoblot analysis (**Figure 2b**).

### Prion strains and Sho down-regulation

**Figure 3:**
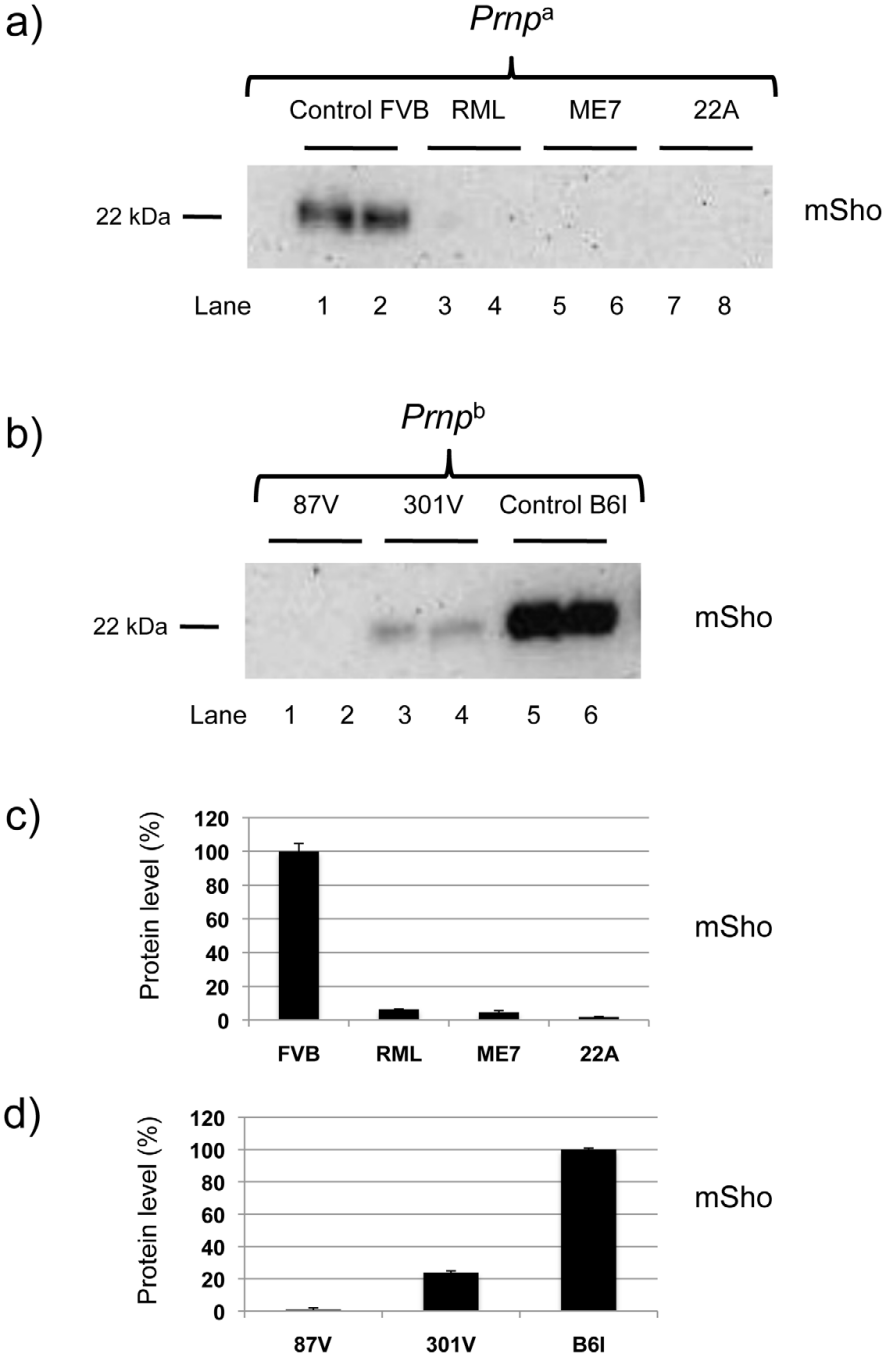
Sho levels at disease endpoint in mice infected with different prion strains. ***a)*** Sho protein western blot analysis of brain homogenates from homozygous *Prnp* ^a^ mice infected with different strain isolates and analyzed at clinical phase of prion disease. Biological replicate samples are analyzed for each permutation (lanes 1 and 2, control inoculum; lanes 3 and 4 RML isolate; lanes 5 and 6, ME7 isolate; lanes 7 and 8, 22A isolate). Analyses were with 06rSH1 antibody. “FVB” and “B6I” represent brain samples from animals inoculated with homogenates from healthy FVB and B6I mice, respectively and, thus, comprise negative controls. ***b)*** Similar analysis to panel (a) performed with mice of the homozygous *Prnp* ^b^ genotype: (lanes 1 and 2, 87 V isolate; lanes 3 and 4, 301 V isolate; lanes 5 and 6, control inoculum). The position of the mature full-length 22 kDa glycosylated Sho protein (mSho) is shown. ***c)*** and ***d)*** Densitometric analysis to show percent reduction in Sho in mice inoculated with different prion isolates as per *a)* and *b)* versus Sho level in control mice with non-infectious inocula.

We next examined prion strains, which are defined as prion isolates with distinct biological properties even when assessed in hosts of the same genetic background. Interestingly, prion strains are often equated with different sub-types of PrP^Sc^ that may be distinguished by parameters such as guanidine denaturation and protease-sensitivity. For these studies we restricted analysis to animals in clinical disease phase. As presented in **Figure 3a**, levels of full-length mature Sho glycoprotein were drastically reduced in *Prnp* ^a^ mice infected with two other isolates of mouse-adapted scrapie prions, ME7 and 22A. *Prnp* ^b^ mice were also used for these analyses. These encode the PrP-B form of PrP^C^, which differs from the A allele at residues L108F and T189V [3], and supports the replication of different prion strains [5]. The “B6I” mice used here derive from repeated backcrosses of the I/LnJ *Prnp* ^b^ allele onto a C57BL6 genetic background to make a congenic inbred strain. 87 V mouse-adapted scrapie and 301 V mouse-adapted BSE agents were assessed in B6I mice (**Figure 3b**). Whereas the 87 V scrapie prion strain isolate also created a strong depression of Sho levels, the BSE-derived 301 V strain [25] had less effect (**Figure 3c, d**).

### Sho is not down-regulated in other neurodegenerative syndromes

**Figure 4:**
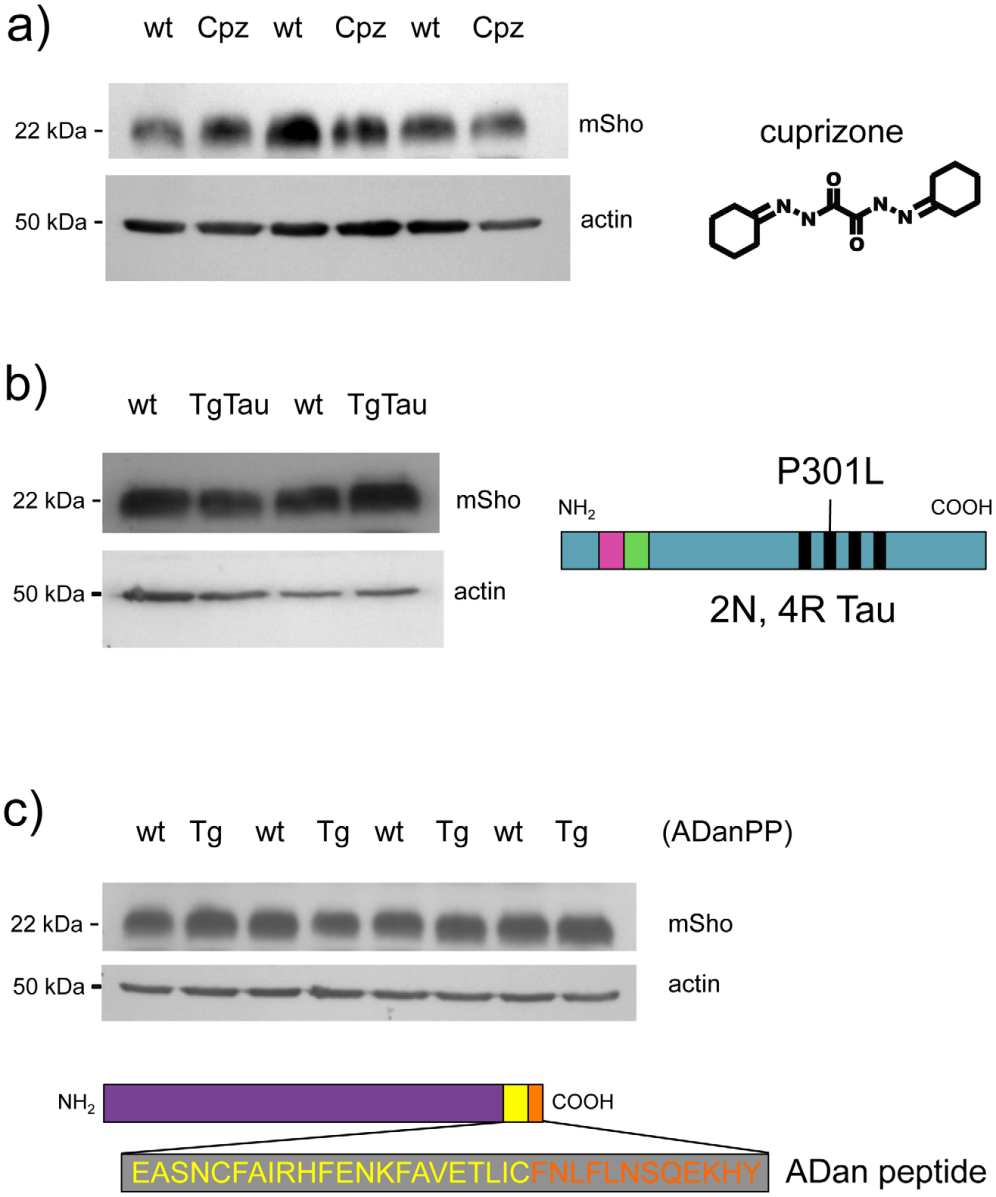
Steady-state levels of Sho protein in mice with ‘non-prion’ neurodegenerative syndromes. Western blot analyses for mature Sho glycoprotein (06rSH1 antibody) are presented for brain homogenates of mice affected by three different disease models. ***a)*** Brains samples from cuprizone-treated animals (Cpz) were analyzed after 8 weeks of dosing (0.4% w/w) alongside non-treated controls (wt, wild type). ***b)*** Tauopathy. Brain samples from non-Tg (wt) and Tg mice of the TgTau(P301L)23207 line (TgTau) [32] were analyzed at age 545 days. Schematic shows the Tau isoform expressed in this transgenic line. ***c)*** Brain samples from 18 month old Tg mice overexpressing the Danish mutant form of *BRI*2 (known as ADan precursor protein; ADanPP) (Tg) and control non-Tg (wt) mice were analyzed by western blot for Sho protein levels. Prior analyses of tissues from these mice have demonstrated pathologic lesions at this age. A schematic of the mutant *BRI2* precursor resulting in extracellular ADan peptide is presented at the bottom of the panel. Wt sequence is represented in yellow while additional sequence present in the mutant is shown in orange. ***a*** **–** ***c)*** In each case normalized protein loading amounts are demonstrated by re-probing with a β-actin-specific antibody (lower panels).

Earlier studies [17] have shown that Sho is not decreased in TgCRND8 mice [26] that are accumulating extracellular deposits of human Aβ peptide. To further assess the issue of disease specificity we used cohorts of animals affected by different neurodegenerative syndromes. In the first instance we used animals dosed with cuprizone, a copper chelator that mimics some histopathological changes apparent in prion infections [27]–[29]. At doses >0.3% cuprizone (weight:weight in food ration), this compound induces progressive demyelination, gliosis, reactive astrocytosis and extracellular vacuolation. At a lower dose, the histopathology is milder with demyelination as the principal change [30]. At the 0.4% w/w dosage used in this study, cuprizone induces prion mimetic changes after 8 weeks of treatment [31]. However, no demonstrable changes were apparent in the steady-state level of Sho protein at the 8-week time-point (**Figure 4a**), suggesting that decrements in protein abundance in prion disease are not merely related to changes in cell populations caused by the proliferation and activation of neuroglia. In the second instance, we examined TgTau(P301L) 23027 mice expressing the “2N, 4R” form of human Tau, which develop a florid 4-repeat Tau tauopathy encompassing CNS neurons, astrocytes and oligodendrocytes [32]. Here in transgenic mice analyzed at 19 months of age, Sho protein levels in the brain were not different from those detected in age-matched non-transgenic littermates (**Figure 4b**). Lastly, we used Tg mice that model the disease Familial Danish Dementia (FDD) by expressing a mutant form of the *BRI2* type II transmembrane protein [33]. These mice start to develop neuropathological lesions by 4 months of age and at a time-point of 18 months these mice exhibit very robust parenchymal and vascular deposition of the amyloidogenic ADan peptide derived from the mutant ADan precursor protein, as well as hemorrhage, microgliosis and reactive astrocytosis [33]. However, at this same 18-month time-point there was no evidence for Sho down-regulation (**Figure 4c**). In sum, reductions in Sho are not part of a generalized response to CNS damage.

### *Sprn* mRNA in different models of infectious prion disease

Levels of *Prnp* mRNA encoding PrP^C^ do not vary during the course of prion infections in hamsters [34], [35]. This finding was verified using microarray analysis of various mouse genetic background/prion strain combinations over the entire incubation period [23], [36]. However, expression of *Sprn* mRNA - which is located on a different chromosome from *Prnp* (chr 7 versus chr 2) [37]-might be quite different.

**Figure 5:**
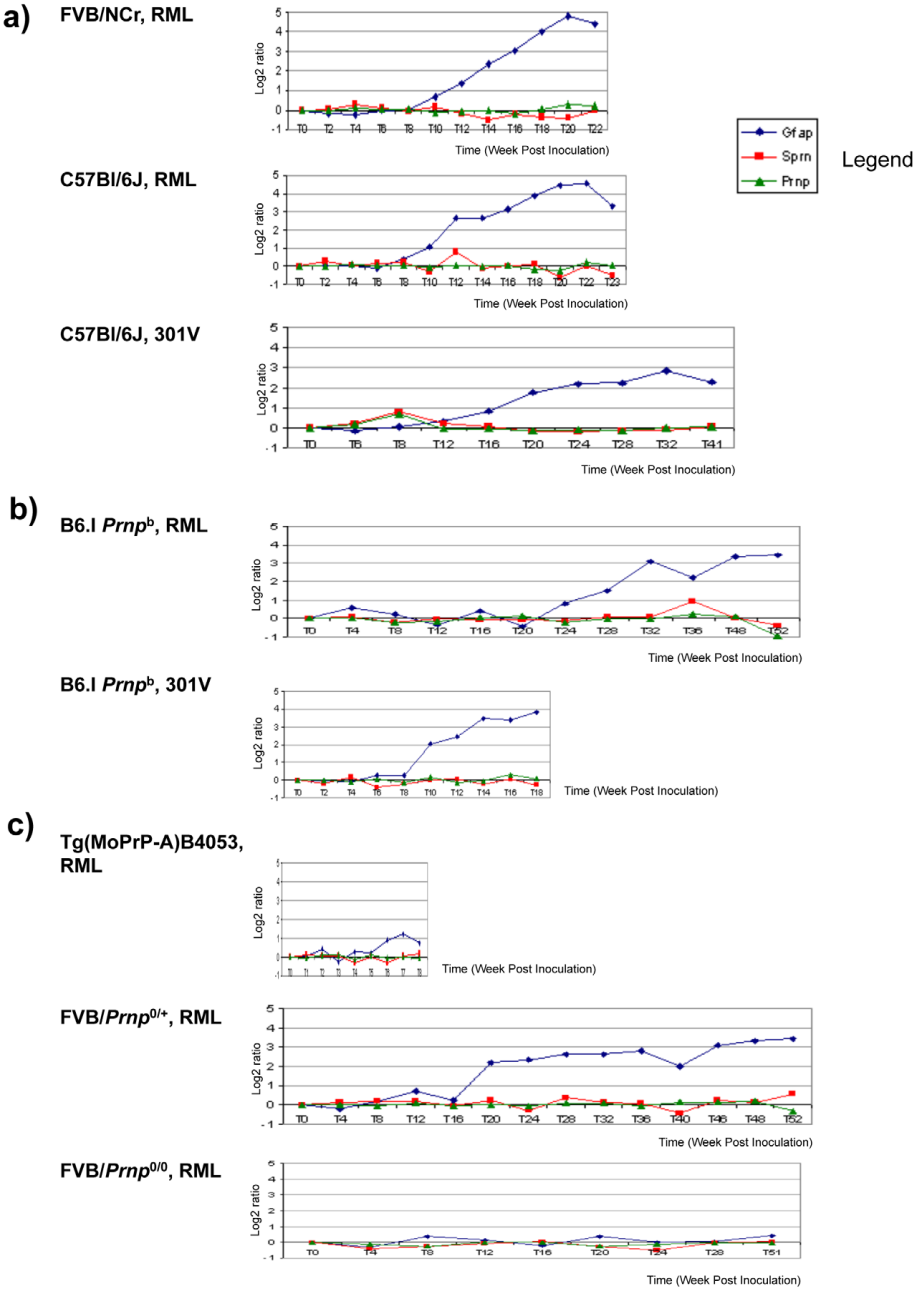
*Sprn* and *Prnp* gene expression time-course in mouse brain. Mice infected with prions show no significant changes for *Prnp* and *Sprn* in three mouse group brains. (**a**) *Prnp^a^* allele-containing mice (FVB, C57Bl/6J) infected with RML or 301 V show almost no changes for *Sprn* and *Prnp*, while showing significant changes for GFAP gene transcripts (“*Gfap”)*; (**b**) Inoculations into *Prnp^b^* mice, C57Bl/6.I-1, show similar trends for those genes with profiles in *Prnp^a^* allele mice; (**c**) other mouse strains with various PrP^C^ concentration deriving from different gene copy-numbers infected with RML: FVB-Tg(PrP-A)4053 (*Prnp* over expressing), *Prnp* ^0/+^ hemizygotes and *Prnp* ^0/0^control mice that are resistant to prion infections. No significant changes were detected for *Sprn* and *Prnp* while significant changes were detected for GFAP only in *Prnp* ^0/+^mouse brain. In each case the x-axis shows the incubation time post-inoculation in weeks and the y-axis shows the gene expression change as the log2 median ratio between prion-infected brain samples and control samples.

Prior analyses have defined 333 differentially regulated core genes (DEG's) that are affected by prion infection [23]. Although *Sprn* was not noted within these initial 333 DEG's, we used this resource to test the hypothesis that down-regulation of Sho protein is a simple consequence of reduced levels of the corresponding mRNA. Thus, we interrogated the Prion Disease Database [36] for *Sprn* mRNA levels within the brains of prion-infected animals and corrected these data versus age-matched animals inoculated with control inocula (‘background subtraction’). *Sprn-*specific probes were included in genome wide microarray analyses performed upon a series of mice of different *Prnp* genotypes infected with one of two prion strains [23] and these data failed to reveal any significant change in *Sprn* transcripts in infected animals (**Figure 5**). Compared with the glial fibrillary acidic protein (GFAP), which is reproducibly modulated by prion infection, only small, inconsistent changes in *Sprn* mRNA abundance were apparent at different time-points. These small alterations in *Sprn* mRNA levels are consistent with experimental noise and cannot explain the 4- to 8-fold decrements in the Sho protein, as documented herein and previously [17], [38]. These microarray data are in agreement with much smaller studies from other laboratories [39] where *Sprn* mRNA levels were sometimes slightly elevated, compared to the Sho protein down-regulation regularly seen in diverse models of prion disease. Thus, reduction in Sho protein in animals with different types of prion infections was not paralleled by a consistent reduction in transcript level.

### Properties of endogenous and transgene-encoded Sho

In a next set of experiments we sought to understand the properties and milieu of the predominant form of wt Sho protein. For example, in cultured cells, beyond internal vesicular compartments and the cell-surface, Sho protein is present in conditioned medium. This suggests “shedding” from the cell-surface, as is the case for PrP^C^ [19], [40], [41]. For biochemical analysis of Sho from brain source material, we used two methods to enrich for membrane-associated cellular fractions. In addition, transgenesis was used to explore the impact of altering the steady-state level of this protein.

**Figure 6:**
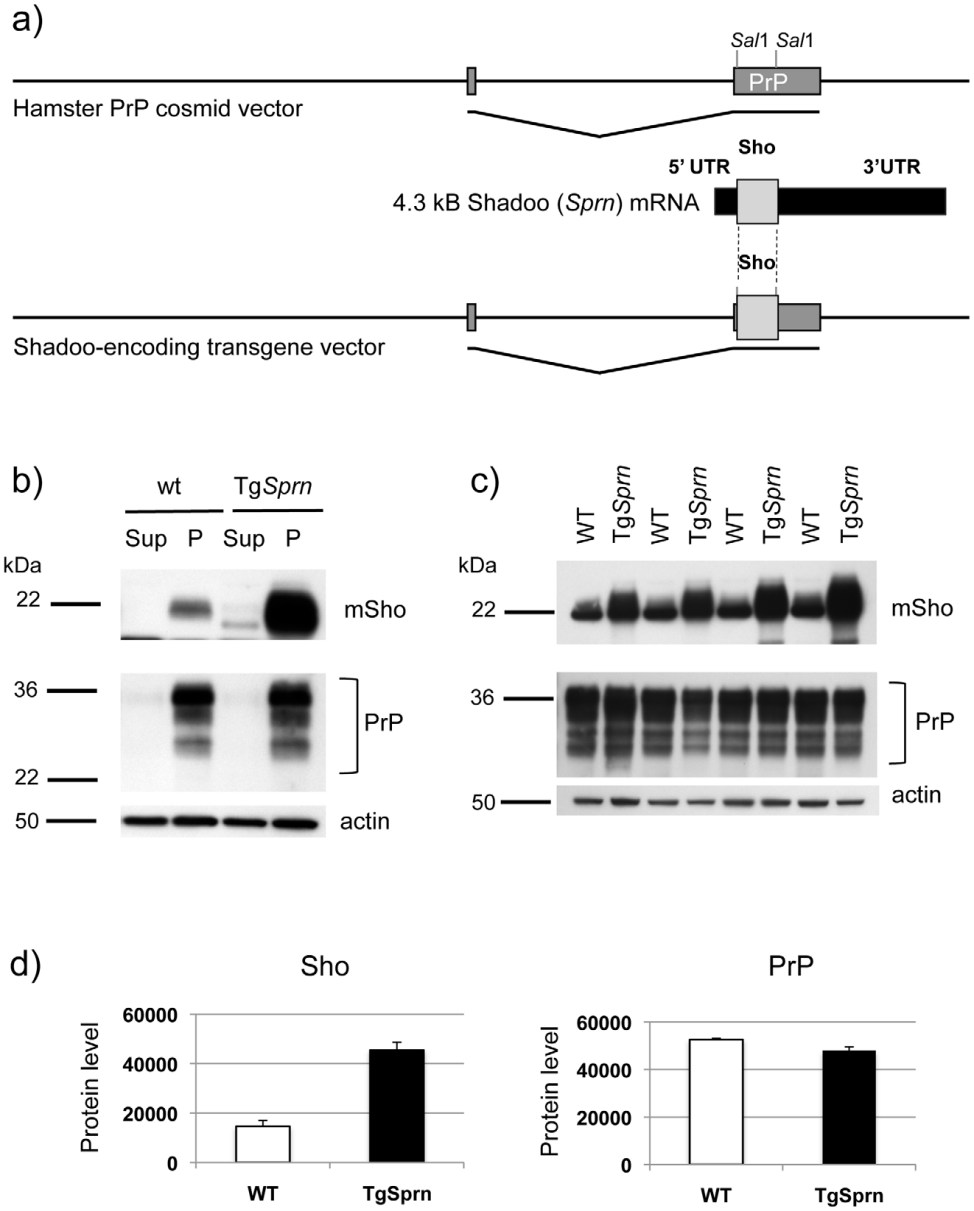
Tg*Sprn* transgenic mice and transgene-encoded Sho protein. ***a)*** Use of the cos.Tet transgene vector to drive Sho protein expression under the control of the Syrian hamster PrP transcriptional unit. A cDNA fragment encoding the wt mouse Sho ORF and devoid of native *Sprn* 5′ UTR and 3′ UTR mRNA sequences was used for this purpose. ***b)*** Upper panel: Blot analysis of supernatant (Sup) and pellet fractions (P) from a 100,000x*g* centrifugation of brain homogenates of wt and Tg*Sprn*24551 mice (N-terminal antibody 06rSH1). Homogenates were prepared in isotonic sucrose. Lower panel: Re-analysis of the same samples with PrP-specific Sha31 antibody indicated a similar pattern of fractionation. ***c)*** Upper panel: Comparison of Sho protein (06rSH1 antibody) levels in a 100,000xg pellet in a litter of Tg*Sprn*24551 (Tg*Sprn*) mice and their non-Tg (wt) littermates. Lower panel: Re-analysis of the same samples with Sha 31 PrP-specific antibody indicates no distinction in the levels of sedimented PrP from Tg and non-Tg animals. Age of analysis of all mice was 95 days. ***d)*** Densitometric analysis of the data presented in panel (c) for Sho and PrP, respectively. Protein level on y-axis is in arbitrary units. The degree of overexpression seen in Tg*Sprn*24551 mice was estimated at 2.5 x.

Tg mice were constructed wherein production of the Sho protein was placed under the control of a heterologous promoter with pan-neuronal expression. For this purpose we used the Syrian hamster *Prnp* transcriptional unit, defined operationally by a ∼42 kB genomic clone comprising the cos.Tet transgene vector [42]. To reduce possible contributions of the *Sprn* 5′ and 3′ mRNA flanking sequences either to translational initiation properties or to mRNA stability, the wt mouse *Sprn* open reading frame (ORF) was mobilized devoid of its native 5′ UTR and 3′ UTR regions (**Figure 6a**), retaining only 12 nucleotides upstream and 1 nucleotide downstream of the ORF, and inserted into the cos.Tet transgene vector. Transgenic mice were produced by standard procedures, resulting in the establishment of one stable line, Tg*Sprn*24551.

**Figure 7:**
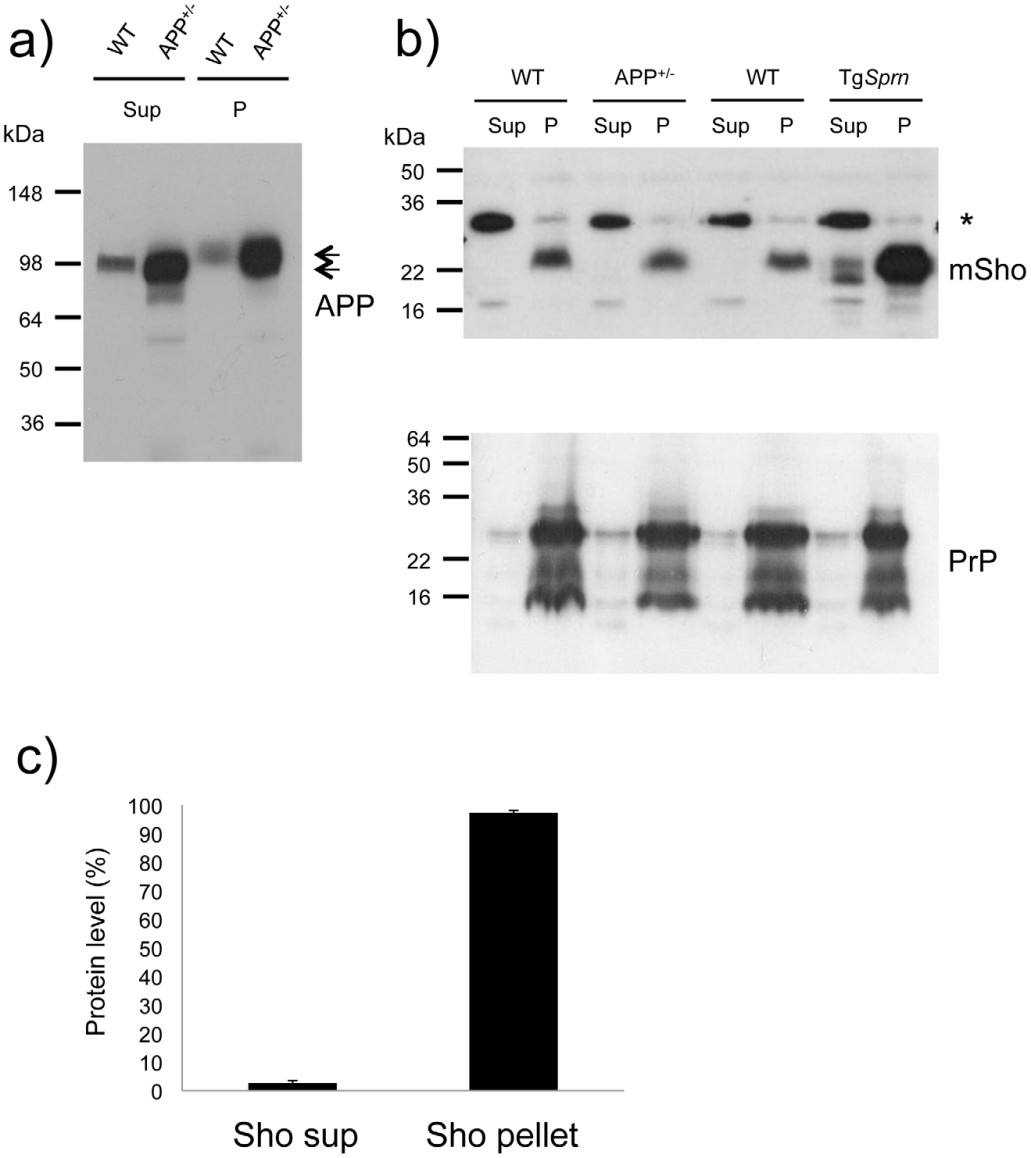
Analysis of DEA extracted APP and Sho from mouse brain. ***a)*** Diethylamine (DEA) extraction for wild type (wt) and TgAPP^+/-^ (APP^+/-^) mouse brains successfully separates the non-integral forms of APP, secreted APP, “sAPP”, as seen in the supernatants (Sup), from the membrane-associated holoprotein that remains in the pellet fraction (P). The TgAPP transgenic line analyzed here corresponds to TgCRND8 mice with ∼ 5x overexpression of APP695 holoprotein. Arrows distinguish the molecular weight difference between processed (extractable) and full-length APP, with electrophoretic mobility of ∼100 and 120 kDa, respectively. ***b)*** Upper panel: Unlike the situation for APP, DEA extraction for wild type (wt) and TgAPP^+/-^ (APP^+/-^) mouse brains fails to release detectable levels of Sho into the supernatant fraction (Sup), and signal remained predominantly in the membrane-associated pellet (P). Lower panel: this demonstrates normalized sample loading by probing for PrP (Sha 31 antibody), with these replica samples being PNGaseF digested to display (from top to bottom) full-length as well as C2 and C1 proteolytic fragments. The asterisk indicates a non-specific band detected with the polyclonal Sho antiserum 06rSH1. ***c)*** Densitometric quantitation of data in (b), upper panel, indicating 97 ±1% SEM of the Sho protein in brain samples of wt mice is present in the pellet fraction. Ages of the presented TgAPP and Tg*Sprn*24551 mice were 8 and 9 months, respectively.

In homogenates of wt mouse brain prepared in isotonic sucrose, most Sho (97%) can be found in a 100,000x*g* pellet, as demonstrated by immunoblotting (**Figure 6b, c**) [17]. The same property was apparent for transgene-encoded Sho, with densitometric analysis indicating overexpression of approximately 2.5×endogenous Sho. PrP^C^ analyzed in parallel was also present in the membrane-enriched 100,000x*g* pellet. The Sho overexpression apparent in Tg*Sprn*24551 mice had no discernable impact on steady-state levels of PrP^C^ (**Figure 6c, d**). In other analyses we employed membrane extraction in alkaline diethylamine buffer (DEA), a technique used previously to distinguish non-integral forms of the amyloid precursor protein (APP), such as APPs-alpha and APPs-beta from membrane-associated mature APP holoprotein [43], [44]. Here, control experiments were performed first to verify separation of APP holoprotein and APP secreted species by this technique, as manifest by a difference in electrophoretic mobility (**Figure 7a**). Sho was then analyzed by the same technique and, as shown in **Figure 7b**, was overwhelmingly associated solely with a membrane-enriched pellet fraction resulting from this procedure, rather than with a supernatant fraction; as demonstrated in **Figure 7c**, this effect was quantitated at 97 ± 1% in the pellet fraction. In Tg*Sprn*24551 mice overexpressing wt Sho the amount of signal within the membrane-associated pellet fraction is greatly increased, as anticipated (**Figure 7b**, upper panel). An immunoreactive signal is also apparent within the supernatant fraction of these Tg mice, perhaps indicating that a small fraction of wt Sho (not detectable in non-Tg animals under the same analytical conditions) is released from cells. Some of the material in this supernatant fraction had a faster electrophoretic mobility than the 22 kDa species in the pellet fractions and analogous species were not seen in equivalent samples from wt mice. These data are potentially consistent with a subset of Sho molecules being cleaved adjacent to the GPI-anchor, as seen here in Tg mice and possibly present in non-Tg mice, though falling below the threshold of detection of the current presented analyses.

The data presented in **Figures 6** and **7** show that the bulk of mature glycosylated Sho in brain samples of wt mice is in a membrane fraction, confirming and extending prior immuno-histochemical staining studies [17].

### Prion disease in Tg*Sprn* mice

**Figure 8:**
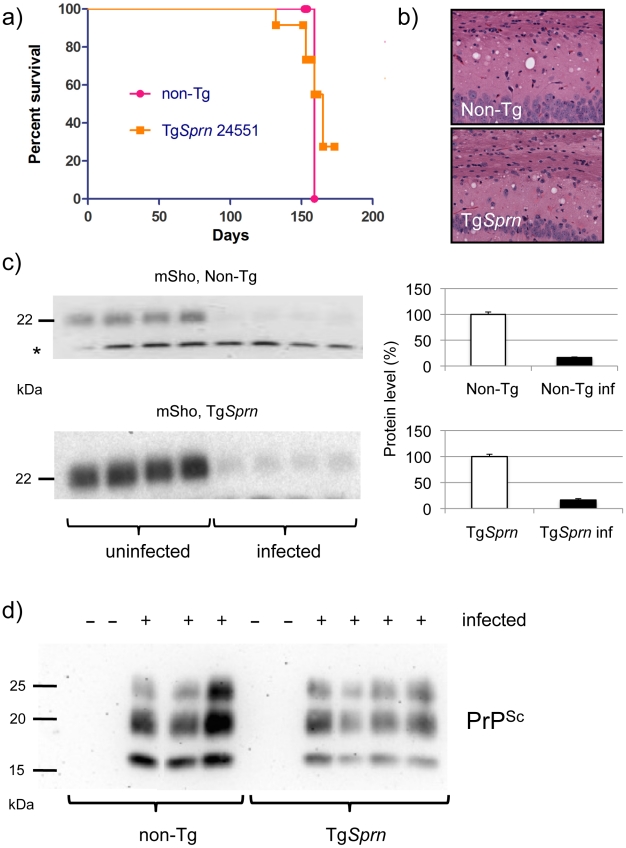
Prion infection assessed in Tg*Sprn* mice. ***a)*** Survival curves of transgenic (Tg*Sprn*) and non-transgenic (Non-Tg) genotypes inoculated with RML prions are presented. Using a log-rank (Mantel Cox) test, the chi-square value was 0.24 and the P value 0.6 (not significant). ***b***) Histopathological analysis of infected animals (hematoxylin and eosin stain of hippocampal neurons and the corpus callosum, intracerebral route of inoculation). Transgenic (Tg*Sprn*) and non-transgenic (Non-Tg) genotypes are shown. ***c).*** Western blot analysis of brain homogenates from groups of four control non-transgenic mice (upper) and four Tg*Sprn* mice (lower) with and without prion infection (RML prion isolate). Animals were in the clinical phase of disease and analysis was performed with the anti-Sho N-terminal antibody, 06rSH1. Bar graphs represent percent remaining in each case, normalized to the uninfected control animals as 100%. The percentage diminution in mSho in both cases was over 90% and with the extent of Sho down-regulation not being significantly different between non-Tg and Tg animals. “inf” signifies infected. A cross-reactive band is indicated with an asterisk and also illustrates similar sample loadings between infected and uninfected animals. ***d)*** Blot analysis of protease-resistant PrP from non-Tg and Tg*Sprn*24551 mice (anti-PrP antibody D18).

Since the Tg*Sprn*24551 line of Sho Tg mice has a) no discernable alteration in the levels of PrP^C^ (**Figure 6b, c** and **Figure 7c**), with PrP^C^ being the obligate precursor to PrP^Sc^, and b) has yet to show signs of spontaneous neurologic disease at ages up to 500 days (n  =  20), it was deemed suitable for prion challenge studies. Accordingly, cohorts of Tg*Sprn*24551 mice were inoculated with RML scrapie prions administered by two different routes. Incubation periods to onset of clinical disease for intracerebral injection were not significantly different between non-Tg and Tg groups (n  =  8, 12). Disease onset was 131 ± 9 (standard deviation) days versus 136 ± 10 days (not significant) and terminal disease was 154 ± 2 days versus 155 ± 10 days (p  =  0.6, ns). Survival curves are presented in **Figure 8a**. For intraperitoneal injection, incubation times to clinical disease for non-Tg and Tg groups (n  =  15, 12) were 163 ± 8 days SD versus 157 ± 9 days (p  =  0.11). Pathology was not notably distinct between the Tg and non-Tg mice infected by the i.c. route (**Figure 8b**). Overall, these data suggest that Sho does not modulate the chemical events that shape the neurologic disease phase of prion infection, in accord with a prior report [45]. However, we demonstrate here, as shown in **Figure 8c**, that levels of Sho protein in transgenic mice were also decreased (p<0.05 infected versus non-infected). The magnitude of this decrease, a ∼90% reduction from control animals to a level of 10.8% ± 1.0 SEM, standard error of the mean, was not statistically distinguishable from that seen in non-Tg mice (also 10.8% ± 1.0 SEM). As noted above, Tg*Sprn*24551 mice overexpress Sho at 2.5x higher levels than wt mice, and insofar as the transgene arrays in these mice are present on a homozygous wt (i.e. *Sprn* ^+/+^) *Sprn* genetic background, this leads to a conclusion that the greater fraction (≥60%, by mass) of the Sho protein in these mice is transgene-encoded. Further, the indistinguishable percentage reduction in Sho between Tg and non-Tg mice data suggests that transgene-encoded Sho is effectively down- regulated. Thus, the down-regulation phenomenon observed in clinical phase of disease in Tg (and wt) mice is likely determined by an intrinsic property of the wt mouse Sho protein.

Blot analyses of protease-resistant protein species were also performed for these cohorts of animals, to ascertain whether the *Sprn* transgene impacted the quantity or quality of PrP^Sc^. These analyses are presented in **Figure 8d** and show a similar pattern of protease-resistant fragments, albeit with a mild decrement in level in the samples from Tg*Sprn*24551 mice.

### Sho and PrP^Sc^ in prion-infected cultures

**Figure 9:**
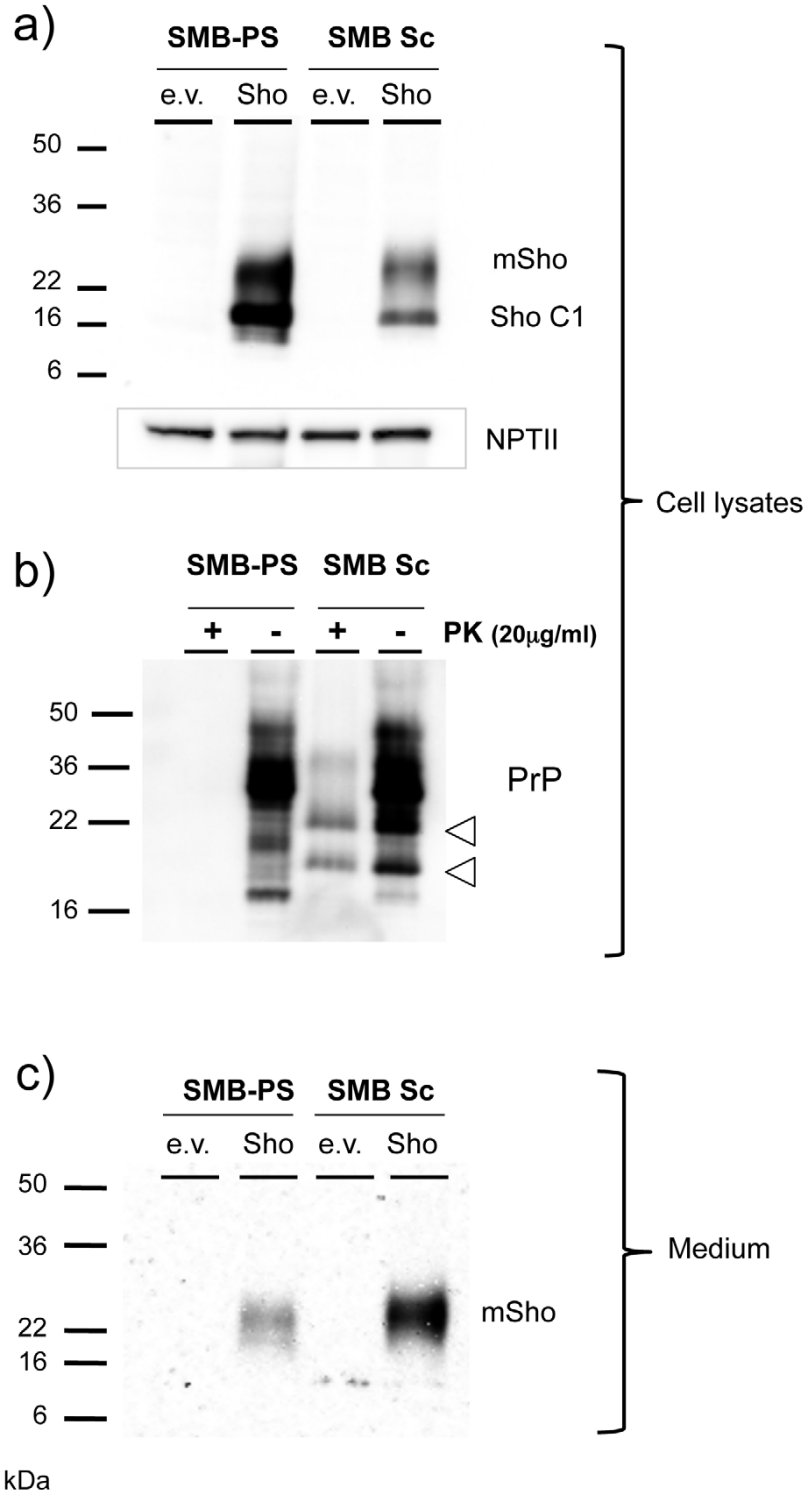
Sho protein in chronically infected SMB cells. ***a)*** Prion-infected SMB cells (SMB Sc) or pentosan sulfate cured SMB cells (SMB-PS) were acutely transfected with a control cDNA expression vector (empty vector, e.v.) or the same plasmid vector bearing a cDNA insert encoding wt mouse Sho (Sho). Upper panel: Cell lysates were prepared from cells 24 hours after transfection and were immunoblotted with a C-terminal anti-Sho antibody 06SH3a. Lower panel: A similar transfection efficiency into healthy and infected cells was established by probing for expression of neomycin phosphotransferase encoded within both varieties of plasmid vector (“NPT II”). C1 is a C-terminal fragment of Sho. ***b)*** The presence of PrP^Sc^ in infected SMB cultures was verified by protease digestion. Two PK resistant fragments co-migrated with fragments present in undigested lysates of infected cells, but not those of uninfected cells (open arrowheads; see also **Figure S1**). ***c)*** Conditioned medium was harvested from the transfected cells, acetone precipitated and then western blotted for the presence of the Sho glycoprotein with 06SH3a antibody.

The protein samples analyzed above derive from homogenates of CNS tissue and are thus uninformative as to the possible contributions of different cell types to the down-regulation phenomenon. However, studies presented thus far suggest that Sho down-regulation is not associated with clinical signs of disease or fulminant pathology in infected animals but, instead, is associated with PrP^Sc^ accumulation. Monocultures of prion-infected cells were used to explore this inference. Such chronically infected cells comprise an experimental paradigm with accumulation of PrP^Sc^ but without (with the exception of GT-1 cells [46]) pathological signs of prion infection. For these experiments we used acute transfection with a Sho plasmid construct devoid of wt 5′ and 3′ mRNA flanking sequences. Using chronically infected SMB cells and non-infected control cells cured by pentosan sulfate treatment [47], the infected SMB cells exhibited net lower levels of transgene-encoded Sho than their non-infected counterparts (**Figure 9a**). Full-length glycosylated Sho detected in cell lysates by a C-terminal antibody was present at 0.58 control level ± 0.16% SEM (n  =  9 experiments). In each of these experiments blot analysis for the vector-encoded neomycin phosphotransferase protein (“α-NPTII”) was used as an internal control for the transfection efficiency of the Sho-encoding plasmid in infected and uninfected cell cultures. Control analyses verified the presence of PrP^Sc^ in the infected cultures (**Figure 9b**), with PK treatment revealing protease-resistant PrP species absent from the control cells. Moreover, in agreement with prior work, two prominent PrP species were apparent in the cell lysates of infected cells (but not in uninfected cells) prior to *in vitro* treatment with PK [48], [49]. Control experiments were consistent with these PrP species deriving from *in vivo* degradation of PrP^Sc^ N-terminal sequences (“N-terminal trimming”) by lysosomal proteases (**Figure S1**).

Since prior experiments demonstrated Sho in the conditioned medium from healthy cultured cells [19], we concentrated this fraction and analyzed Sho protein species by western blot analysis. These analyses revealed that Sho species recovered from conditioned medium of infected cells often, but not always, (5/9 analyses) demonstrated a slower electrophoretic mobility than equivalent samples derived from healthy cells (**Figure 9c**). In addition, mean levels of Sho protein in the medium normalized for transfection efficiency were often but not always higher than in uninfected cells, but with variance greater than observed for the cell-associated fraction (2.5 ± 1.0 fold higher, mean ± SEM). Taken together, these data support the notion that Sho levels are reduced in the prion-infected SMB cell monocultures. However, the maximum degree of reduction observed in cell-associated Sho was less than in infected brain - 40% reduction, versus experiments with ≥ 95% reduction in brain samples from late-stage disease, as analyzed here (or previously, [38]).

## Discussion

### Sho protein as a marker of prion infections

The generation of α-Sho antibodies has allowed an appreciation that down-regulation of the mature full-length form of Sho glycoprotein is a consistent feature of prion infections using different cohorts of animals infected with the RML isolate of rodent-adapted scrapie prions [17]. As demonstrated by our analyses, the potent drop in Sho protein level seen with the RML isolate of prions (and different prion strains, below) does not comprise a generalized response to CNS damage, as it is absent from other experimental paradigms of neurodegenerative diseases analyzed in their ‘late’ or ‘end-stage’ phases. Thus, Sho protein is not depleted in Aβ-depositing mice with synaptic damage and hippocampal memory deficits [17], [26], [50], in cuprizone treated mice [31], in mice with a 4-repeat tauopathy [32] and in mice expressing the mutant *BRI2* protein of familial Danish dementia [33] (**Figure 4**). Instead, we conclude that down-regulation of Sho is a prion disease-specific event.

In kinetic analyses, we (**Figures 1** **,** **2**) and others [51], have analyzed time-courses of prion infections and we demonstrate here that down-regulation of Sho and clinical prion disease are not synchronized. This effect is most dramatic in infected *Prnp* hemizygous animals (**Figure 2a**), where PrP^Sc^ accumulation rises to a plateau hundreds of days before the onset of clinical disease. What is apparent instead is an inverse relationship between the steady-state levels of full-length 22 kDa Sho protein and sub-clinical accumulation of protease-resistant PrP. Since this Sho depletion does not synchronize with clinical disease, we can emphatically exclude an earlier hypothesis that Sho modulates clinical signs of prion infection via a mechanism where neuroprotective activity engendered by Sho becomes reduced as the full-length protein is down-regulated [17].

### A protease hypothesis for Sho depletion

The amount of wt Sho is not correlated with *Sprn* transcript levels, either in a variety of prion strain types or in mice of different PrP genotypes (*Prnp* ^+/+^ versus *Prnp* ^0/+^; **Figure 5**). Also, Sho protein encoded by mRNAs with heterologous mRNA 5′ and 3′ flanking sequences (i.e. in Tg*Sprn* mice using hamster *Prnp* 5′ and 3′ UTRs) is still subject to down-regulation (**Figure 8**). These data argue against a regulatory mechanism involving the Sho mRNA, or translational initiation signals encoded therein. Our cumulative analyses indicate a relationship whereby the degree of down-regulation of the mature Sho protein is related in an inverse fashion to the levels of PrP^Sc^. This consistent biochemical relationship (**Figures 1b** **,** **2a, 2b**, [51]) occurring at the preclinical phase suggests the existence of an underlying biological process. In addition, beyond observations of protein levels in Tg*Prnp*, *Prnp* ^+/+^ and *Prnp* ^0/+^ mice, we have also undertaken studies wherein levels of Sho are manipulated genetically. Mice with more Sho (Tg*Sprn*) do not have notably altered neurologic signs or neuropathology (**Figure 8a, b**), indicating that the wt mouse Sho protein is not a disease-modifier.

Our data point to a posttranslational mechanism for reductions in Sho protein level. A process of elimination and the following observations suggest a key role for proteolysis. We note that a) PrP^C^ is an obligate precursor to PrP^Sc^ [6], [52], b) Sho and PrP^C^ occupy similar membrane environments (**Figures 6** **,** **7**) [53] with neuroanatomical overlaps in expression [17] and c) Sho is more protease-sensitive than PrP^C^. Sho lacks a folded C-terminal domain and, by available criteria, is natively unstructured *in vivo* [17]. Sho from N2a cells and mouse brain is degraded by PK concentrations of 0.5 µg/ml, approximately 10-fold lower than those used to degrade PrP^C^ from the same sources [19]. We hypothesize that production of PrP^Sc^ is sensed within the CNS, or in infected cultures, and that this triggers a cellular response to degrade this molecule. In this scheme, Sho residing in close proximity to PrP^C^ and the sites of templated misfolding would then be degraded via a ‘bystander’ effect, i.e. as an “inadvertent” substrate for this cellular response. Since Sho is reduced by up to 90% in prion infections (**Figure 8c**) and since 97% of Sho resides in a membrane-associated pellet fraction as determined by two techniques (**Figures 6** **,** **7**), we infer that the bulk, membrane-associated form of Sho would be the substrate for the down-regulation effect. In the framework of this hypothesis, Sho expressed at endogenous levels, or overexpressed 2.5-fold, is efficiently degraded by the activity. Hence in the situations described here, as PrP^Sc^ rises, so Sho falls.

It remains possible that reductions in Sho levels are not due to a proteolytic mechanism directly targeting PrP^Sc^, but are triggered by other host responses to prion replication, perhaps involving a protein degradation pathway distinct from that for PrP^Sc^. Reasons why this option is less likely are presented elsewhere in the Discussion, but future experiments deploying selective protease inhibitors will be necessary to solidify our present concept.

### Sho down-regulation and prion strains

As discussed above Sho down-regulation is a robust phenomenon applying to many prion strain isolates *in vivo*. However, the degree of down-regulation observed in the clinical phase of disease may vary with different prion isolates [38], the latter being characterized by different levels of accumulation of protease-resistant PrP^Sc^ (although none of the six strain isolates analyzed by Miyazawa *et al* coincide with those used here). In our studies (**Figure 3b, d**) and analogous studies described by Watts *et al.* [51], one prion strain, 301 V, stands out with only a modest decrement by clinical phase of disease. This strain is a mouse-adapted form of BSE with a distinctive pattern of neuroanatomic lesions and has a heightened tropism for a *Prnp* ^b^ genetic background [1], [5]. Although not analyzed in *Prnp* ^b^ mice, a modest reduction of 40% in Sho levels was reported for a mouse-adapted vCJD isolate with a predominant representation of di-glycosylated forms of PrP [**38**]. Thus it will be important for future studies of Sho down-regulation to correlate percentage reduction with prion physiochemical properties defined by techniques beyond PK digestion.

### Protease pathways for depletion of Sho?

Protein clearance systems pertinent to neurons such as the ERAD-proteasomal pathway, lysosomes and autophagosomes are candidates for mediating this process, but comprise generic cellular mechanisms for handling diverse types of accumulating proteins. While a ‘bulk-flow’ mechanism cannot be excluded, e.g. wherein Sho is physically trafficked with PrP^Sc^ and hence processed by one of the generic protease systems, this would require a new hypothesis to be invoked where Sho associates selectively with PrP^Sc^, but not with misfolded extracellular proteins such as Aβ or ADan.

In our studies Sho down-regulation was not present in three other forms of protein misfolding disorders, these being the cytoplasmic accumulation of Tau in Tg(P301L)23027 mice [32], and parenchymal and vascular accumulation of Aβ and Bri peptide in TgCRND8 and TgADanPP7 mice, respectively [26], [33], [51]. In a similar manner, while astrocytes and microglia react to protein accumulations (and in the latter case can perform phagocytosis), these properties are shared throughout a number of diseases. In the context of the disease models used here, TgCRND8 and TgADanPP7 mice exhibit robust reactive gliosis and microgliosis [26], [33]. Similar observations pertain to cuprizone toxicity [54], with both reactive astrocytosis and microgliosis seen, and with GFAP activation used as a point of reference in microarray analyses (and reaching a plateau at the 8-week time-point analyzed here) [31]. For Tg(P301L)23027 mice, 4-repeat Tau accumulates inside astrocytes and manifests as astrocytic plaques and is also present in oligodendrocytes [32], yet no alteration in Sho level was seen in pathology-age transgenic animals (**Figure 4b**).

Interestingly PrP^Sc^ half-life has been measured at ∼1.5 days via the use of regulated PrP^C^-encoding transgenes [55], although the mechanism responsible for this clearance was “unknown”. At this time cell-culture assays with potential logistic advantages for mechanistic studies of Sho regulation (e.g. acute dosing with protease inhibitors) have a restricted dynamic range: ∼2x, versus up to ∼10x in brain (**Figure 9** and [51]). Reasons for the restricted effect size in infected cells may include the acute nature of the assay, or the lower titre of prions attainable per unit volume in homogenates of cultured cells versus brain homogenates [56], [57]. We also note that while lysosomes are notably activated in prion infected cells - to the extent that PrP^Sc^ is already trimmed at the N-terminus without the need for PK digestion *in vitro* (**Figure 9b**, **Figure S1**; [48], [49]) – these infected SMB cells have a smaller effect size for Sho down-regulation than that seen in infected brain samples. These data do not galvanize support for a key role of lysosomal pathways in Sho regulation, and raise the issue of whether lysosomal systems are a confound for robust cell-culture studies.

While acquiring the identity of the prion degradative pathways is an important future goal, several inferences can nonetheless be drawn from the present hypothesis. First, if a protease system is activated in preclinical prion infections then there must be a sensing system for misfolded prion proteins. While there has been recent interest in a ubiquitin-coupled system for handling misfolded polytopic transmembrane proteins [58], a system capable of handling extracellularly disposed GPI-linked proteins would be of considerable interest. Second, net output of a prion-directed proteolytic system could depend on the stoichiometry and protease-resistance of any competing substrates. As noted, mouse Sho is natively unstructured and susceptible to digestion [19] such that we hypothesize it as a “tracer” rather than a competitive inhibitor. However, there is natural variation in Sho sequences [59], [60] and, in one instance, recombinant allelic forms of Sho protein have different proteinase sensitivity [19]. Two UK patients heterozygous for an *SPRN* frameshift allele were found in a sample of 107 vCJD cases, and a signal peptide sequence missense polymorphism was overrepresented in cases of sporadic CJD [61]. While human Sho may behave differently from mouse Sho (as the protein is implied by genetic criteria to be a disease modifier rather than a disease tracer) it is possible that pathogenic effects of human Sho alleles might emanate from altered interactions with, or sensitivity to, prion disease protease systems.

## Materials and Methods

### Ethics statement

The studies at McLaughlin Research Institute (MRI), which is fully accredited by AAALAC International, were carried out in accordance with the Guide for the Care and Use of Laboratory Animals of the National Institutes of Health, U.S. Public Health Service. MRI's Animal Assurance number from the Office of Laboratory Welfare of the National Institutes of Health is A3901-01. All procedures involving animals were reviewed and approved by MRI's Institutional Animal Care and Use committee under protocol GAC-05. Intracranial inoculations were performed under isoflurane anesthesia and every effort was made to minimize discomfort to the mice.

### Animal resources, prion inoculations and clinical diagnosis

TgCRND8 mice [26] were maintained on an outbred C3H/C57BL/6 strain background, Tg4053 [62] and Zrch 1 *Prnp* ^0/0^ mice [63] were maintained on an FVB/N background, while B6I (*Prnp* ^b^) [62] and TgTau(P301L) 23027 mice [32] were maintained on a congenic C57BL/6 background. TgADanPP mice were as described previously [33]. Prion inoculations (20 µl of 1% brain homogenate) and clinical diagnoses were done as described previously [21], [62]. Prion strain stocks (RML, ME7, 22A, 87V, and 301V) were sourced from S. Prusiner, University of California San Francisco, USA. Cohorts of inoculated *Prnp* ^a/a^, *Prnp* ^0/+^ and Tg.*Prnp* ^a^ mice have been described previously [23] and supporting data can be accessed at: http://prion.systemsbiology.net. Methods for formalin fixation, paraffin embedding and staining of prion-infected tissue were as described previously [64].

### Cuprizone treatment

Bis(cyclohexanone)oxaldihydrazone, cuprizone, (Sigma Aldrich, St. Louis, MO) was formulated with 6% fat rodent chow (Teklad, Madison, WI) to a final concentration of 0.4% w/w. Ten week-old male C57BL/6 mice were fed cuprizone or control chow for 4 or 8 weeks, a dose at which no mortality was observed. Subsequently, animals were euthanized, brains were removed and tissue was homogenized to 10% w/v in PBS with protease inhibitors (Roche, Indianapolis, Ind).

### Construction of TgSprn mice

A RPCI-22 mouse BAC clone of 129 mouse genome DNA (TCAG Genome Resource Facility, Hospital for Sick Children, Toronto, Canada: BAC clone 274D12) was digested with *Eco*RI endonuclease and the resulting 15 kb fragment containing *Sprn* genomic sequences was cloned into the plasmid vector pBR322. This plasmid was then used as a template for PCR amplification using primers that contained *Sal*I sites (5′-ttttgcgtcgacCTGCCCAGTAGGATGAAC-3′ and reverse primer 5′-ga agg gtc gac TCT AAG GCC GAA GCA GTT C-3′). The PCR products were cloned in pTOPO2.1 plasmid (Invitrogen), and sequenced to confirm the absence of PCR errors. The resulting plasmid was digested with *Sal*I to release a fragment that was cloned into Cos.*Fse*.Tet [42], [65]. *Fse*I transgene fragments excised from this cosmid vector were separated by gel electrophoresis, extracted by the freeze-and-squeeze method [66] and injected into oocytes of FVB/NCr background. Founder animals were identified by dot-blot hybridization analysis of genomic DNA using a probe within the hamster PrP gene 3′-untranslated region, as described previously [67].

### Immunoaffinity Purification of Polyclonal Anti-Sho Antibody 06rSH1

An affinity column consisting of recombinant GST-Sho(25-80) covalently attached to glutathione-Sepharose 4B (GE Healthcare) by coupling reaction with dimethyl pimelimidate (DMP, Thermo Scientific) was used to capture 06rSH1 [17] from rabbit immune serum. The serum was loaded onto the column and re-circulated for 2 h at 4°C, followed by washes with PBS containing 0.5 M NaCl and 1 mM EDTA and then PBS-EDTA alone. The antibody was then eluted with 0.1 M glycine-HCl (pH 2.5) and immediately neutralized by collection into one-tenth final volume of 11M Tris-HCl (pH 8). Recovery of antibody was monitored by western blotting against recombinant Sho and mouse brain homogenate. The purified 06rSH1 antibody was dialyzed against PBS-EDTA and stored at −20°C.

### Chronically-infected SMB cells

Uninfected and infected SMB cells [47], [68], [69] were grown in DMEM complete medium. Cells were seeded in 60 mm Petri dishes and transfected with Sho expression vector plasmids using Lipofectamine (Invitrogen). Conditioned media was collected 24 hours after transfection and acetone precipitated. After centrifugation, pellets were re-suspended in gel loading buffer.

### Isotonic sucrose centrifugation

10% brain homogenates prepared in 0.32 M sucrose containing complete protease inhibitor cocktail (Boehringer) were spun at 100,000x*g* for 1 h at 4°C and pellets re-suspended in cold 50 mM Tris-HCl, pH 7.5, 0.5% Triton X-100, 0.5% deoxycholate, and a protease inhibitor cocktail. 50 µg of total protein sample was used for western blot analysis.

### Diethylamine extraction

Diethylamine (DEA) extraction of APP and Sho from mouse brains was performed by a method previously described [43], [44]. Briefly, brains were homogenized 10% w/v in a solution containing 50 mM NaCl and 0.2% DEA. Homogenate was ultracentrifuged at 100,000 xg for 1 h at 4°C in a bench top ultracentrifuge (Optima MAX, Beckman, Fullerton, CA). The resulting supernatant was neutralized with the addition of 0.5 M Tris-HCl pH 6.8 at a volume one-tenth that of the total. Pellets were re-suspended in buffer containing 50 mM Tris (pH 7.5), 0.5% sodium deoxycholate, and 0.5% Triton X-100 by serially passing through hypodermic needles ranging in size from 16 to 21 gauges. For western blot analysis, an equal amount of each sample (12.5 µg for APP and 50 µg for Sho) was separated on a Tris–Glycine SDS-PAGE gel (8% for APP and 14% for Sho).

### Protein blot analyses

SMB cells were either directly analyzed by SDS-PAGE or digested 20 min at 37°C with Proteinase K (PK) (20 µg/ml), followed by addition of 2 mM PMSF. Infected brain homogenates were digested 1 h at 37°C with PK (1 µg/50 µg of total protein) followed by addition of 2 mM PMSF. Histoblots were performed as described previously [70] and with some histoblots reproduced with permission from the prion disease database [36]. Antibodies used were for Sho: Anti C-terminus 06SH3a and anti N-terminus Sho 06rSH1 [17]. For PrP: SHA31 (Medicorp Inc.), 8B4 (a generous gift from Dr. Man-Sun Sy) and D18 [71]. NPTII (Millipore) was used for control of cell transfection. APP A4 (Millipore) was used for the detection of APP. GAPDH and β-actin antibodies were obtained from Sigma.

### Statistical analysis

Survival curves were analyzed by Log-rank (Mantel-Cox) test (Microsoft Excel) and variance in protein levels was analyzed by GraphPad for Mac version 3.1a. Alpha value for significance was set at 0.05.

## Supporting Information

Figure S1 **N-terminal trimming of PrP^Sc^ in chronically-infected SMB cells.** To confirm that novel PrP species present in undigested samples from prion infected cells (open arrowheads: see also Figure 9b) represented the effects of lysosomal proteases, cells were plated without (***a***) or with (***b***) NH_4_Cl for 5 hours (30 µM). Protein samples were detected with Sha31 recognizing a C-terminal epitope in PrP helix 1. In contrast, an N-terminal antibody 8B4 did not reveal PrP species that were distinct between undigested lysates derived from control and infected cells.
(TIF)
