# Supplementary figures and images for: Down-Regulation of Shadoo in Prion Infections Traces a Pre-Clinical Event Inversely Related to PrPSc Accumulation
Source: PLoS Pathog. 2011 Nov 17;7(11):e1002391. doi: 10.1371/journal.ppat.1002391 (PMC3219720; doi:10.1371/journal.ppat.1002391)

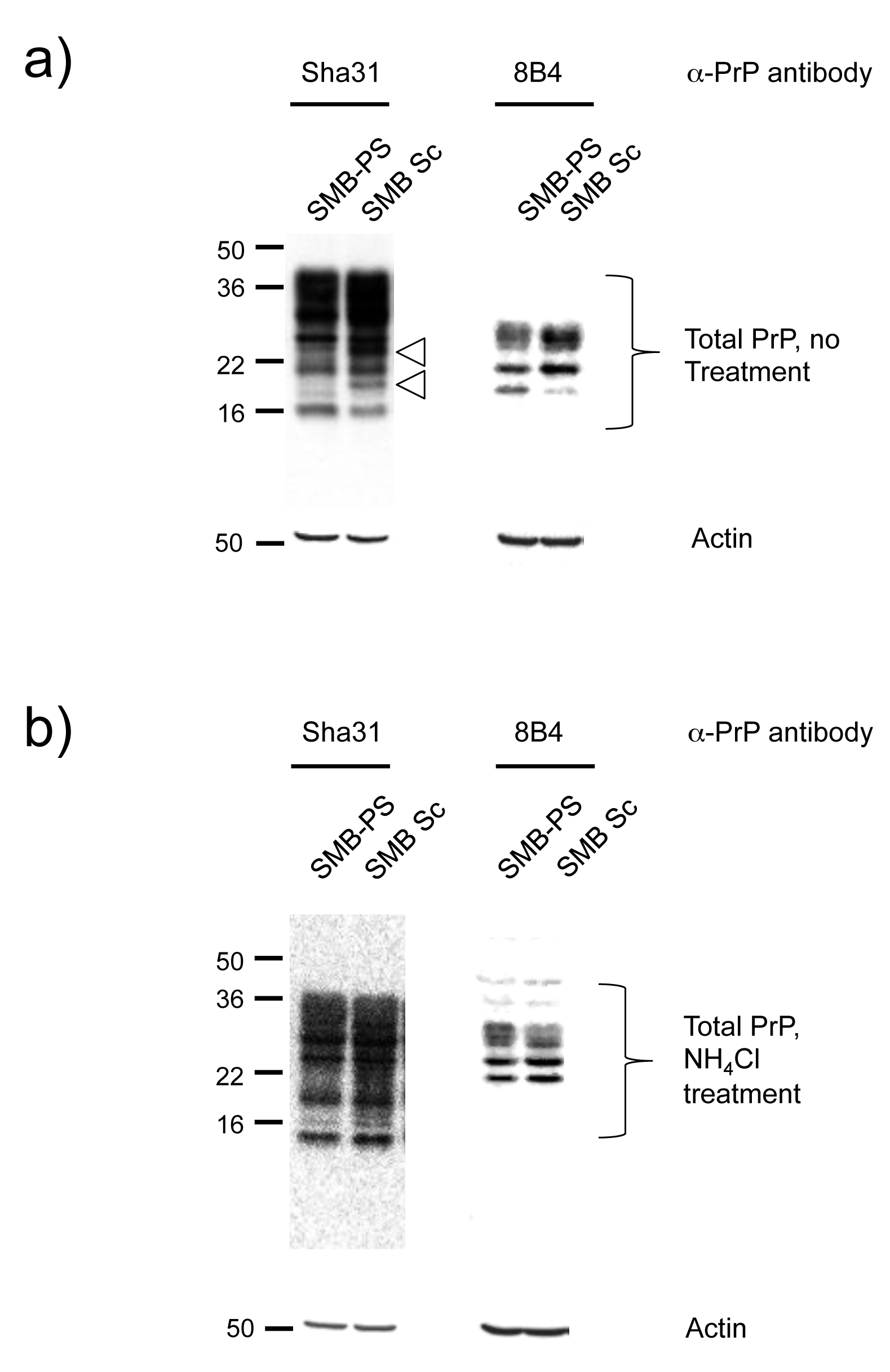

Supplement: Figure S1 — N-terminal trimming of PrPSc in chronically-infected SMB cells. To confirm that novel PrP species present in undigested samples from prion infected cells (open arrowheads: see also Figure 9b) represented the effects of lysosomal proteases, cells were plated without (a) or with (b) NH4Cl for 5 hours (30 µM). Protein samples were detected with Sha31 recognizing a C-terminal epitope in PrP helix 1. In contrast, an N-terminal antibody 8B4 did not reveal PrP species that were distinct between undigested lysates derived from control and infected cells. (TIF) [file ppat.1002391.s001.tif]
